# Applying the Stages of Change Model in a Nutrition Education Programme for the Promotion of Fruit and Vegetable Consumption among People with Severe Mental Disorders (DIETMENT)

**DOI:** 10.3390/nu13062105

**Published:** 2021-06-19

**Authors:** Mireia Vilamala-Orra, Cristina Vaqué-Crusellas, Quintí Foguet-Boreu, Marta Guimerà Gallent, Ruben del Río Sáez

**Affiliations:** 1 Research Group on Methodology, Methods, Models and Outcomes of Health and Social Sciences (M3O), Faculty of Health Sciences and Welfare, University of Vic—Central University of Catalonia, 08500 Vic, Spain; mvilamala@osonament.cat; 2 Osonament—Osona Psychopedagogical Medical Center Foundation, 08500 Vic, Spain; guimeramarta7@gmail.com (M.G.G.); rdelrio@osonament.cat (R.d.R.S.); 3 Multidisciplinary Research Group in Mental Health, Faculty of Medicine, University Hospital of Vic, University of Vic—Central University of Catalonia, 08500 Vic, Spain; qfoguet@chv.cat; 4 Research Group on Mental Health, University of Vic—Central University of Catalonia, 08500 Vic, Spain; 5 Social Innovation (SaMIS), University of Vic—Central University of Catalonia, 08500 Vic, Spain

**Keywords:** mental disorders, mental health recovery, fruit and vegetables consumption, nutrition education, health behaviour, transtheoretical model

## Abstract

Despite growing evidence of the benefits of adequate intake of fruit and vegetables (F&V) and the recommendation to consume five servings daily, the adoption of these habits is poor among people with severe mental disorder (SMD). The main aim of the present study is to determine changes in the intake of F&V and motivation to do so among people with SMDs after participating in a food education programme. A community-based randomized controlled trial was conducted in Spain, with the intervention group (IG) participating in a food education programme based on the stages of change model to promote consumption of F&V and the control group (CG) receiving three informative sessions on basic healthy eating. The main outcomes were related to the intake of F&V and stages of change. Data collection was performed at baseline, post intervention, and 12-month follow-up. Seventy-four participants enrolled in the study and sixty completed the 12-month follow-up. An increase in motivation towards the intake of F&V was observed in the IG but not in the CG (McNemar’s test *p* = 0.016, *p* = 0.625). No significant difference was observed for the intake of fruit, vegetables, or F&V. Basing food education strategies on the stages of change model shows positive results, increasing the awareness and disposition of people with SMD towards the intake of F&V. More research is needed to identify the most appropriate eating intervention to increase the intake of F&V.

## 1. Introduction

Following a diet rich in fruit and vegetables (F&V) is associated with numerous health benefits [1,2], not only in the prevention of non-communicable diseases such as cardiovascular disease [3] and certain types of cancer [4], but also increased well-being and quality of life, as well as a positive impact on mental health, reducing levels of anxiety, stress, and depressive symptoms [5,6,7].

The World Health Organization (WHO) recommends a minimum intake of 400 g of F&V a day as part of a healthy diet, prioritizing the consumption of fresh fruit and emphasizing the importance of variety [8]. These recommendations have been adopted and promoted through national institutions such as the Catalan Public Health Agency and the Spanish Society of Community Nutrition, which recommend including at least five servings of F&V a day [9,10].

In Catalonia, only 14% of people over the age of 15 consume five or more servings of F&V a day [9]. A low intake of these food groups has also been identified in other regions, such as the United States and European Union countries [2,11]. Evidence of the consumption of F&V is even lower among people with severe mental disorders (SMDs) [12]. Various studies have described a poorer dietary pattern among people with SMDs, characterized by a high intake of total energy, saturated fat, and sugars and a low intake of fibre, fruit, and vegetables, along with other unhealthy eating styles, including disordered eating habits, fast-eating syndrome, and increased consumption of junk food [13,14].

SMDs encompass a set of mental disorders of different natures and clinical presentations, displaying common criteria in terms of severity and persistence over time, with a tendency towards deterioration and alteration of social, family, and work functioning, thus, reducing the individual’s quality of life [15].

The adoption of unbalanced eating habits is considered to be one of the individual risk factors in the accumulation of metabolic disorders among people with SMDs, such as obesity, diabetes mellitus, and metabolic syndrome, resulting in a high presence of cardiovascular comorbidity [16,17]. Cardiovascular disease is identified as the most common cause of death in this group [18]. People with SMD have significant clinical complexity, not only due to the mental disorder, but also to the associated medical comorbidities, increasing the risk of premature death [16]. It is a group that is vulnerable to various chronic health problems, such as the aforementioned cardiovascular diseases, respiratory tract diseases, infectious diseases (HIV), and cancer, with a proportion of disease that doubles that of the general population, and with the consequent impact on their quality of life [18].

However, the adoption of unhealthy eating behaviours is identified as a modifiable risk factor, which makes it an opportunity for intervention [19]. Developing programmes to promote healthy eating, and in particular the consumption of F&V, aimed at people with SMDs, encourages the voluntary adoption and maintenance of eating habits that promote the health and well-being of the individual. Most food education programmes assume that the individual starts out with a certain degree of willingness and awareness to change behaviour, and therefore focus the intervention on action-oriented activities, predicting abandonment for those individuals not yet ready to see it through [20].

The transtheoretical model [21] views behavioural change in health as a gradual, continuous, and dynamic process that takes place in a series of stages based on the individual’s willingness to adopt the change. The model establishes five stages: (1) precontemplation—a lack of intention with regard to short-term behavioural change; (2) contemplation—ambivalence stage, the need for behavioural change is considered but not in the immediate future (next 6 months); (3) preparation—a behavioural change approach is adopted in the near future (next 30 days); (4) action—obvious changes have been made aimed at achievement of the desired behaviour; and (5) maintenance—modifications made are maintained for more than six months. At the same time, the model postulates that there are two mediators of change, i.e., decisional balance (pros and cons) and self-efficacy, and ten processes that modulate change, understood as experiential or cognitive and behavioural strategies that people use to progress in different stages [22].

Several studies have recognized the relevance of basing interventions on the stages of change model, allowing for strategies to be designed that respond to the needs of participants, and thus effectively facilitating the adoption of healthy behaviour [23,24]. Although several authors have designed and implemented intervention strategies that have been aimed at people with SMDs and focused on increasing the practice of physical activity and improving diet quality, no studies have yet been described that employ this model to promote healthy eating behaviours [25]. 

The main aim of the present study is to investigate changes in the intake of F&V and motivation to do so among people with SMDs after participating in a food education programme based on the stages of change model. In addition, we also investigate the association between the intake of F&V and non-motivational factors (sociodemographic and clinical variables) and describes the disposition towards the intake of five daily rations of F&V according to the aforementioned model.

## 2. Materials and Methods

### 2.1. Study Design and Participants 

A randomized community-based controlled trial was conducted between January 2019 and September 2020. The sample (*n* = 74) consisted of individuals with SMDs who were also participants at the Osonament community mental health services, a comprehensive care service offering a network of resources to cover needs related to recovery, housing, leisure, and employment for people with mental health problems and addictions in the region of Osona (Catalonia, Spain).

The following inclusion criteria were defined: individuals over the age of 18 with a clinical diagnosis of SMDs who were active users of Osonament services, and who according to DSM-IV TR [26] had received a diagnosis of schizophrenia or other psychotic disorders, bipolar disorder, major depression, obsessive-compulsive disorder, or personality disorders. The following individuals were excluded: users of the residential services, those diagnosed with substance use disorder, dementia, relapse of mental disorder, moderate to severe intellectual development disorder, and individuals receiving a specific dietary treatment with contraindication for the consumption of F&V.

The original sample required 52 people per group to allow a detectable difference between groups, with an expected 20% percentage change in the proportion of participants who achieve the intake of five daily servings of F&V, considering a significance of 0.05, 80% power in a unilateral contrast, and estimating a follow-up loss rate of 10%.

Once the baseline data had been collected, participants were randomly assigned to the IG (Dietment programme) or CG using the Zenon algorithm (equiprobable randomization 1:1 through R Software), considering the variables of age, gender, functionality, and primary mental health diagnosis. A researcher otherwise unconnected to the study performed the randomization.

All participants, and legal guardians if required, gave their consent for inclusion before participating in the study. The study was conducted in accordance with the Declaration of Helsinki, and the protocol was approved by the Clinical Research Ethics Committee (CEIC) belonging to the Osona Foundation for Health Research and Education (FORES) in September 2018 (CEIC code 2018974).

### 2.2. Intervention 

The Dietment intervention programme lasted four months (April to July 2019). It consisted of a food education strategy aimed at promoting the consumption of F&V and comprised 15 group sessions (of 5–10 people) lasting 90 min each session, which were held weekly. All sessions were conducted by the same dietitian-nutritionist.

The intervention design was based on the stages of change construct included in the transtheoretical model [21]. Considering that this was an intervention group and assuming that the participants would be at different stages of readiness with regard to the intake of F&V at baseline, the content of the sessions was sequenced gradually according to the five stages of change throughout the programme (Table 1). All IG participants completed the 15 sessions of the programme, regardless of their motivation to change at baseline.

The food recommendations provided were taken from the guide to healthy eating prepared by the Catalan Public Health Agency [9]. No food guide has yet been identified for people with mental disorders.

### 2.3. Measures 

Data were collected at baseline (T0) and once completed the intervention (T1) for both groups (IG and CG). To determine the long-term effect, it was repeated 12 months after the end of the intervention (T2). A questionnaire was used to re-collect the sociodemographic and clinical data as well as the different study variables.

#### 2.3.1. Sociodemographic Characteristics

Sociodemographic variables included age, gender, marital status, level of education, and whether support was available for carrying out activities of daily living (ADL).

#### 2.3.2. Clinical Characteristics

The mental health diagnosis was obtained from the medical history. At the anthropometric level, the current weight and height were collected, making it possible to obtain the body mass index (BMI) [27]. The Functioning Assessment Short Test (FAST) [28] was used to determine functionality, the Spanish version of the Screen for Cognitive Impairment in Psychiatry (SCIP-S) [29] was used for cognitive impairment, and the Spanish version of the Personal Well-Being Index was used for subjective well-being [30].

#### 2.3.3. The Consumption of F&V

Data on the consumption of F&V were collected by means of two questions related to: “serving of fruit consumed the day before” and “serving of vegetables consumed the day before”. The answers to the two questions were added together to obtain the total consumption of F&V. The interviewer explained which foods were included in the two groups, as well as what constituted the consumption of a serving of F&V.

#### 2.3.4. Stages of Change

Classification into each of the stages was determined using the algorithm proposed by Laforge et al. [31]. This is a two-step algorithm consisting of four possible questions, which allows individuals to be placed in one of the five stages proposed in the transtheoretical model of stages of change about their readiness to intake five servings of F&V.

As described by the authors, participants were asked about the number of servings consumed the day before: if the answer was five or more daily servings, they were asked if the behaviour had been maintained over more than six months. Participants who responded from 0 to 4 servings were asked about the intention to eat a minimum of five servings a day in the next six months. If they responded “yes”, they were asked whether they intended to eat a minimum of five servings per day over the next 30 days.

Respondents were assigned to the precontemplation stage if they reported usually eating fewer than five servings of F&V daily and had no intention of changing their behaviour. The contemplation stage was defined as usually eating fewer than five daily servings and reporting the intention to adopt the change within the next six months. People were assigned to the preparation stage if they reported intending to adopt the change within the next 30 days. People were considered to be in the action stage if they reported usually eating five or more servings of F&V but had been doing so for less than six months. The maintenance stage was defined as eating a minimum of five servings daily for more than six months.

Participants’ disposition towards switching to the consumption of F&V was not taken into account for recruitment or randomization purposes.

For the analysis of paired data, the stages of change variable were re-coded to a dichotomous variable grouping the stages of pre-contemplation, contemplation and preparation (pre-action) and action and maintenance (action/maintenance).

### 2.4. Statistical Analysis

A descriptive study was carried out, the categorical variables being described using absolute frequencies and percentages, and the measures of central tendency (mean, median) and dispersion (standard deviation (SD), percentiles) being calculated for the quantitative variables. The assumption of normality of the continuous variables was studied, as well as the total scores of the tests used.

An analysis of independent samples was performed between the two randomization groups to study whether the groups were homogeneous a prior (baseline). The comparison for categorical variables was performed using Pearson’s chi-square test or Fisher’s test if appropriate. The comparison of means was carried out by means of a Student’s *t*-test.

The Pearson’s correlation coefficient, Anova, and Student’s t-test were used to analyse the association between consumption of F&V and the different sociodemographic and clinical variables. The Anova and post hoc tests were used to determine the association between the stages of change and the intake of F&V.

Given the small size of the study sample, non-parametric techniques were applied (Friedman and Wilcoxon signed-rank test) to study the evolution in the number of rations of F&V consumed between baseline, post, and 12-month follow-up evaluations and for the two intervention groups. The Mann–Whitney U Test was applied to detect a difference between groups at baseline (T0), post intervention (T1), and 12-month post intervention (T2). The McNemar test was applied to determine the effect of the intervention on disposition towards change, based on the comparison of response percentages prior to and after the intervention.

## 3. Results

### 3.1. Sociodemographic and Clinical Characteristics

**Figure 1:**
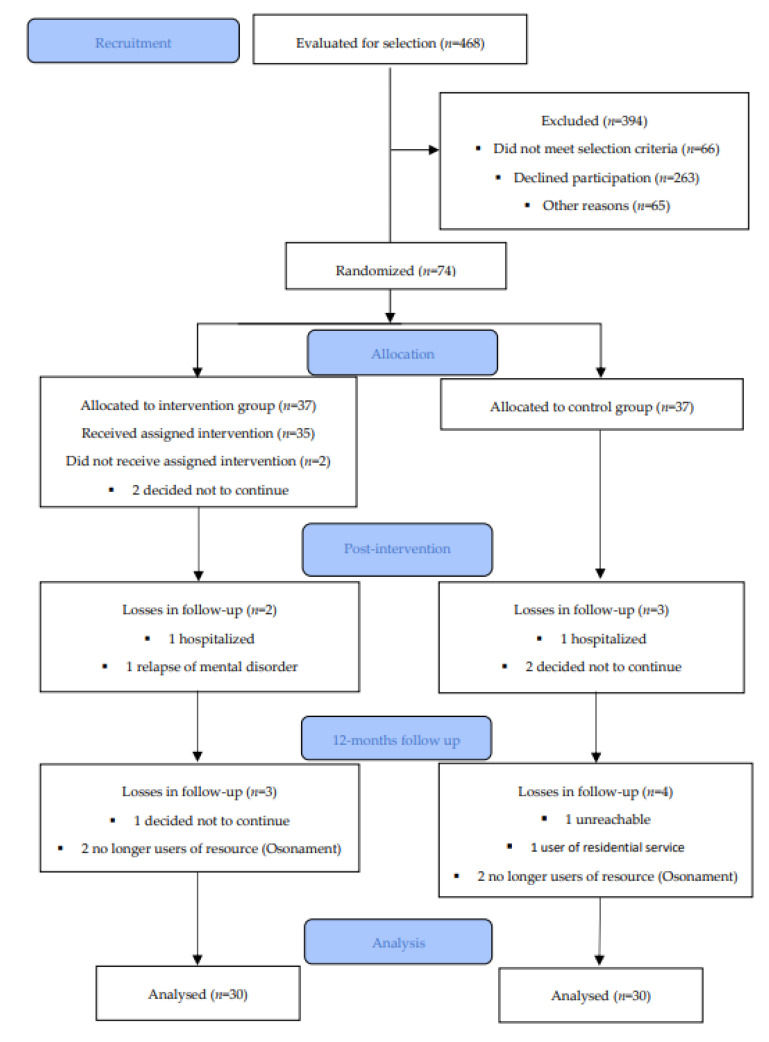
Participation flow chart (CONSORT 2010).

Seventy-four users of the Osonament community services completed baseline data collection, 37 of whom were assigned to the IG and 37 to the CG. The original target sample size could not be reached. Complete data could be collected for 67 people at the end of the intervention (T1), and for 60 people one year after its completion (T2). Figure 1 shows the flow of participation throughout the study. A per-protocol analysis was performed. The losses in follow-up were reported equally in both randomized groups and in a staggered manner throughout the study. The intention-to-treat analysis was not performed due to the impossibility of assigning values to the missing cases.

The mean age was 48.7 years (SD 10.8), the majority (55.4%) were men. The most common diagnosis of mental health was schizophrenia and other psychotic disorders (37.8%). At baseline, the groups were homogeneous in terms of sociodemographic and clinical characteristics (Table 2). Although there were no statistically significant differences between the two groups with regard to BMI, it is worth noting that the IG was above the threshold for obesity while the mean for the CG fell within the category of overweight.

### 3.2. Sociodemographic and Clinical Characteristics Related to the Intake of F&V

Table A1 and Table A2 present the results of the relationship between the different sociodemographic and clinical variables and the intake of F&V, collected at baseline and for the total sample (*n* = 74). No statistically significant associations were found with age, gender, marital status, level of education, primary diagnosis of mental health, weight, BMI, functionality, cognitive impairment, or subjective well-being. A significant association was only observed between the average serving of F&V consumed and support received for ADL: 3.2 (SD:1.5) in individuals receiving professional support, 1.9 (SD:1.7) in those not receiving support, and 1.8 (SD:1.6) in those receiving family support (*p* = 0.022).

### 3.3. Association between Stages of Change and the Intake of F&V 

**Figure 2:**
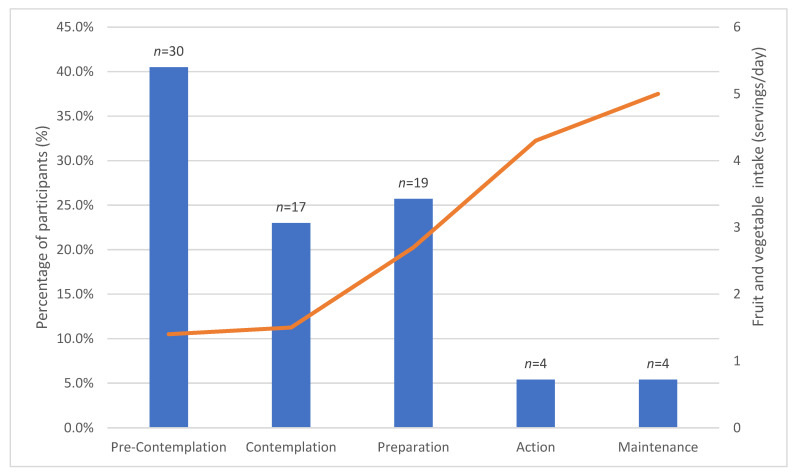
Daily intake of F&V according to stages of change.

Figure 2 details the distribution of participants among the different stages of change and the consumption of F&V according to these stages at baseline (*n* = 74). Some 63.5% of participants were in pre-contemplative or contemplative stages, and 10.8% reported following the defined behaviour (action and maintenance). Table 3 shows the mean daily intake of F&V by stage of change, with an overall significant difference between stage of change and the intake of F&V (*p* < 0.001). Those participants in the action/maintenance stages reported a significantly higher intake of F&V than those in the pre-contemplation and contemplation stages.

### 3.4. Consumption of F&V at Baseline and Impact of the Intervention

At baseline (*n* = 74), 10.7% of participants reported consuming the recommended amount of F&V (five servings/day) (5.4% of the IG participants and 16.2% of the CG participants), the median number of servings consumed being two (IQR 2.3). Some 82.4% ate fewer than three servings of fruit and 82.4% ate fewer than two servings of vegetables. At post intervention (*n* = 67), 15% of participants achieved five daily servings of F&V (21.3% of the IG participants and 8.8% of the CG participants).

No statistically significant differences were observed for the intake of fruit (*p* = 0.124), vegetables (*p* = 0.110), or F&V (*p* = 0.595) between the IG and the CG at baseline (*n* = 74). The IG showed a significance increase in the consumption of fruit and F&V over time (between T0 and T1 *p* = 0.007, *p* = 0.005), but no difference was observed in the CG (*p* = 0.086). No differences were observed in relation to vegetable intake in the two randomized groups over time (Table 4). Comparing the IG and CG at post intervention and 12-month follow-up, no statistically significant differences were observed in the consumption of fruit, vegetables, and F&V. 

### 3.5. Impact of Intervention on Motivation to Change

Between baseline and post intervention, 23.3% of the IG participants progressed to the action/maintenance stage (*p* = 0.016), while the change was not significant for the CG (*p* = 0.625).

**Figure 3:**
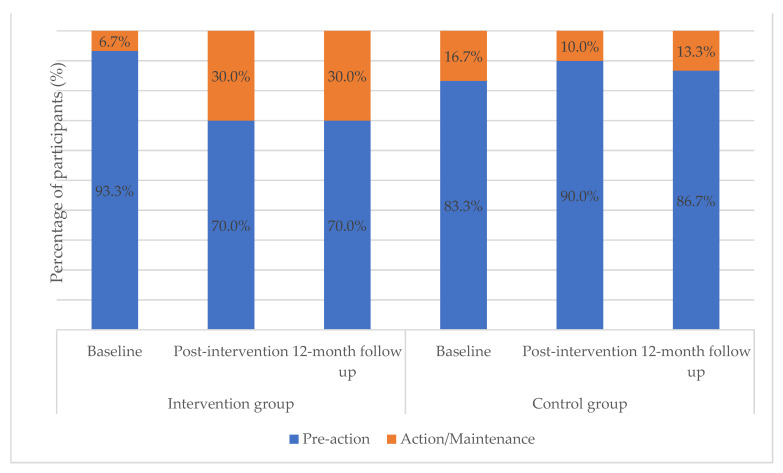
Evolution in the stages of change according to assignment group (*n* = 60).

Analyzing the change between post intervention and 12-month follow-up (T1 and T2), no significant differences were observed in either of the groups. Figure 3 shows the percentage of individuals who were in each stage at different times.

## 4. Discussion

A dietary education intervention based on the stages of change model to promote consumption of F&V among people with SMDs was found to increase the number of people who switch to action/maintenance stages by 23%. Despite the observation of an increase in fruit intake over time by the IG, the change was not significant as compared with that of the CG. This study did not find any significant difference in the intake of vegetables and F&V between the groups over time, highlighting the challenges of promoting healthy eating habits among people with SMD.

At baseline, only 10.7% of the sample studied achieved the recommended intake of F&V, with an overall average consumption far below the five recommended daily servings of F&V. Various studies have shown the consumption of F&V to be below the established recommendations among people with mental disorders [13]. In a Spanish sample of people diagnosed with schizophrenia, Simonelli-Muñoz et al. [32] found that 91% consumed fewer than four daily servings of F&V. 

There are many factors that can condition and influence food choices [33]. Although different studies have sought to determine non-motivational factors associated with the intake of F&V, few have explored this in people with SMDs. Our results reveal an association between receiving professional support for autonomy in ADLs and higher intake of F&V, while no other variable showed such an association.

Other authors have identified a relationship between gender, age, and BMI and the intake of F&V [12]. In a qualitative study that addressed the factors that condition the acquisition of healthy eating habits conducted with people with SMDs and care professionals, both groups pointed to the complexity of maintaining healthy eating habits when professional support is no longer in place, often leading to an abandonment of acquired behaviour [34].

Our study indicates that at baseline almost half of the participants (47%) received professional or family support to carry out basic self-care activities and instrumental activities independently. However, it should be noted that such support does not always encourage healthy eating habits, as the results show the consumption of F&V to be below the minimum recommended intake. Thus, receiving support is identified as a favourable aspect in the adoption and maintenance of healthy eating behaviours, with a positive impact on the quality of the diet, as long as this environment promotes health and the people who are responsible for eating follow a healthy eating pattern themselves [35]. 

With regard to the disposition of the sample studied towards increasing the intake of F&V, most participants were in stages of non-disposition; 64% reported being pre-contemplative or contemplative at baseline, denoting a low awareness of the consequences of insufficient intake of F&V, while those in the pre-action stages were found to display a lower intake of F&V as compared with those in the action stages. On the basis of a qualitative exploration of barriers to adopting healthy eating in adults with mental health problems, Barre et al. observed that most were in stages of pre-contemplation or contemplation, and that those who adopted dietary changes towards a healthier eating pattern were motivated by a perception of personal risk after an episode of illness [35]. Studies of an overweight population and a university population have shown that a greater disposition towards the consumption of F&V is related to a higher intake [36,37].

To date, several authors have designed and implemented interventions to promote healthy lifestyles aimed at people with mental disorders [38]. Despite this, few studies have developed food education strategies in this group with the aim of empowering individuals to adopt healthy eating patterns and encourage the maintenance of changes over time. Use of the stages of change model to design interventions that promote healthy lifestyles and, in particular, the consumption of F&V, is supported by numerous studies, showing positive results in terms of increasing intake. However, these prior interventions have been aimed primarily at children and young people in general [39,40]. 

Our results show that using the stages of change model to design interventions for promoting healthy eating aimed at people with SMDs returns favourable results with regard to increasing motivation for the consumption of F&V. Basing the intervention design on the transtheoretical model allows for the use of specific intervention strategies that increase in effectiveness with each stage, providing a series of defined strategies to facilitate movement towards stages of greater disposition towards the desired behaviour [41]. Although much evidence was found regarding the effectiveness of using this model to promote a change in eating behaviour, significant results were not observed with regard to increasing the intake of F&V.

Limitations of the present study include: the small sample size may limit the power of the study in detecting differences between groups, while it may also condition uneven sampling at different stages of change; collecting information on the intake of F&V that is only based on consumption in the previous 24 h can present a bias in determining actual intake—a prospective 72-hour record or a record of non-consecutive days could also be included; there is a possible risk of information contamination between the participants of the IG and the CG, due to their attending the same centre and being located in the same environment; and finally, the period between post intervention and 12-month follow-up coincided with the COVID-19 pandemic, although there is insufficient evidence of this exerting a possible influence on the results obtained.

The strengths of the study include its design, which allowed for comparison with a control group, and follow-up of participants at 12 months post intervention. In addition, the inclusion of programmes for promoting healthy eating habits in community rehabilitation services as an integral part of the individual’s recovery process, and the evolution of individuals’ disposition towards acquiring healthy eating behaviours. Although the sample size can be considered small at the statistical level, taking into account the target profile, it is concluded that there has been a high participation and adherence to the programme, with less than 20% loss one year after the end of the intervention.

## 5. Conclusions

Despite growing evidence of the benefits of an adequate intake of F&V, people with SMDs who participated in this study displayed a consumption that was far removed from the established recommendations of five daily servings. This highlights the need to develop food education programmes that facilitate the disposition towards changing behaviour and promoting the intake of F&V among individuals in these groups. Despite there being a trend to increase fruit intake among the IG, this result was not statistically significant as compared with the CG. Basing food education strategies on the stages of change model yields positive results in relation to an increase in the disposition of people with SMDs towards a higher intake of F&V. More experimental studies are needed on the effectiveness of behavioural interventions aimed at changing eating habits in people with SMDs.

## Figures and Tables

**Table 1:** Applying the stages of change model in design of the intervention (Dietment Programme).

Stages of Change	Session	Objective	Process of Change	Session Content
Pre-contemplation	1st, 2nd session	Raise awareness of the need for change	Consciousness-raising Dramatic relief	Debate on beliefs and attitudes towards healthy eating (1st session) Identify facilitating elements and barriers to habit change (1st session) Present news items on the health impacts of adopting unhealthy eating behaviours (1st session) Role of emotional state in eating behaviours and food selection (2nd session)
Contemplation	3rd, 4th, 5th session	Motivate and increase confidence in the ability to change	Consciousness-raising Self-reevaluation	Self-reevaluation of current eating habits via a dietary record (24 h reminder) (3rd session) Presentation and debate about false beliefs related to food (4th session) Benefits of the consumption of F&V (4th session) Workshop on the amount of sugar in foods and healthy alternatives (5th session)
Preparation	6th, 7th session	Work on knowledge and skills	Self-liberation	Individual assessment of possible modifications to be introduced in eating habits (6th session) Set specific short-term goals (6th session) Seasonality of F&V and strategies to increase their intake (7th session) Suggestions for healthy breakfasts and snacks (7th session)
Action	8th, 9th, 10th, 11th, 12th, 13th session	Increase self-confidence and enhance autonomy (skills training)	Counterconditioning Stimulus or environmental control	Cooking workshop (servings and recipes) (8th and 13th session) Producing a healthy recipe book according to the season (9th session) Creating an individual diet plan (9th session) Workshop on purchase planning (10th session) Food remaining management workshop (11th session) Disposable tablecloth design with messages promoting consumption of F&V (12th session)
Maintenance	14th, 15th session	Work on social support Preventing relapse into unhealthy eating behaviours	Environmental re-evaluation Helping relationships	Self-assessment of current eating habits and comparison with dietary record completed at the beginning of the programme (14th session) Sharing experiences (peer support) (15th session) Group sessions aimed at relatives and flat/housemates

**Table 2:** Baseline sociodemographic and clinical characteristics of participants.

Characteristics	Total Population (*n* = 74)	IG (*n* = 37)	CG (*n* = 37)
Age (years), mean (SD)	48.7 (10.8)	49.8 (11.4)	47.7 (10.3)
Gender, *n* (%)			
Men	41 (55.4)	21 (56.8)	20 (54.1)
Women	33 (44.6)	16 (43.2)	17 (45.9)
Marital status, *n* (%)			
Single	46 (63)	19 (52.8)	27 (73)
Separated or divorced	18 (24.7)	13 (36.1)	5 (13.5)
Married or has a partner	9 (12.3)	4 (11.1)	5 (13.5)
Level of education, *n* (%)			
No schooling	1 (1.4)	1 (2.7)	-
Secondary	32 (43.2)	12 (32.4)	20 (54.1)
Upper secondary or VT	31 (41.9)	19 (51.4)	12 (32.4)
Further education	10 (13.5)	5 (13.5)	5 (13.4)
ADL support, *n* (%)			
No support	39 (52.7)	19 (51.4)	20 (54.1)
Family or non-professional	20 (27)	10 (27)	10 (27)
Professional	15 (20.3)	8 (21.6)	7 (18.9)
Main diagnosis, *n* (%)			
Schizophrenia and other psychotic disorders	28 (37.8)	12 (32.4)	16 (43.2)
Bipolar disorder	22 (29.7)	12 (32.4)	10 (27)
Depressive disorders	19 (25.7)	10 (27)	9 (24.3)
Personality disorders	3 (4.1)	1 (2.7)	2 (5.4)
Obsessive-compulsive disorder	2 (2.7)	2 (5.4)	-
Weight (kg), mean (SD)	84.2 (18.1)	87.1 (17.5)	81.3 (18.5)
BMI (kg/m^2^), mean (SD)	29.6 (6.3)	30.6 (5.6)	28.7 (6.8)
FAST, mean (SD)	34.4 (13.1)	32 (14)	36.8 (11.9)
SCIP-S (PT), mean (SD)	36.3 (7.7)	37.1 (8.1)	35.5 (7.3)
Subjective well-being, mean (SD)	57.3 (19.3)	53.8 (19.6)	61.1 (18.6)

IG, intervention group; CG, control group; VT, vocational training; ADL, activities of daily living; BMI, body mass index; FAST, Functioning Assessment Short Test; SCIP-S, Screen for Cognitive Impairment in Psychiatry. No differences were observed between participants according to randomization group (*p* > 0.05).

**Table 3:** Mean servings of F&V by stage of change.

Stage of Change	F&V	*p*-Value ^1^	*p*-Value ^2^				
	Mean (SD)		Pre-Contemplation	Contemplation	Preparation	Action	Maintenance
Pre-contemplation	1.4 (1.7)	<0.001	-	1.0	0.031 *	0.004 *	<0.001 *
Contemplation	1.5 (1, 0)		1.0	-	0.092	0.007 *	<0.001 *
Preparation	2.7 (1.4)		0.031 *	0.092	-	0.279	0.034 *
Action	4.3 (0.9)		0.004 *	0.007 *	0.279	-	0.945
Maintenance	5 (1.4)		<0.001 *	<0.001 *	0.034 *	0.945	-

F&V, fruit and vegetables; ^1.^ Anova; ^2.^ Post-hoc test; * *p* < 0.05.

**Table 4:** Changes in the intake of F&V in each allocation group.

	Baseline (T0) (*n* = 60)	Post Intervention (T1) (*n* = 60)	12-Month Follow-up (T2) (*n* = 60)	*p* ^1^	Difference from T0 to T1 *p*-Value ^2^	Difference from T1 to T2 *p*-Value ^2^
Result	Median (P25–75)	Median (P25–75)	Median (P25-75)			
IG						
Fruit	1 (0–2)	2 (1–3)	2 (1–3)	0.012 *	0.007 *	0.088
Vegetables	1 (0–2)	1 (0–2)	1 (0–2)	0.219	0.432	0.251
F&V	2 (1–3.3)	3 (2–4.3)	3 (2–4)	0.081	0.005 *	0.447
CG						
Fruit	1 (0–2)	2 (1–2.3)	1 (0–2)	0.086	0.098	0.095
Vegetables	1 (0–1)	1 (0–1)	1 (0–2)	0.439	0.527	0.653
F&V	2 (0.8–3)	3 (1.3–3)	2 (1–4)	0.237	0.212	0.243

P25–P75, Percentile 25—Percentile 75; IG, intervention group; CG, control group; F&V, fruit and vegetables; ^1.^ Friedman’s test; ^2^ Wilcoxon signed-rank test; * *p* < 0.05.

## Appendix A

**Table A1:** Association between sociodemographic and clinical variables and F&V intake.

Characteristics	Servings F&V	*p*-Value
	Mean (SD)	
Gender		
Men	1.9 (1.8)	0.319 ^a^
Women	2.3 (1.7)	
Marital status		
Single	1.9 (1.7)	0.365 ^b^
Separated or divorced	1.6 (1.8)	
Married or has a partner	2.3 (1.9)	
Level of education		
No schooling	1	0.841 ^b^
Secondary	2.2 (1.8)	
Upper secondary or VT	2.1 (1.6)	
Further education	1.8 (2.1)	
ADL support		
No support	1.9 (1.7)	0.022 ^b,^*
Family or non-professional	1.8 (1.6)	
Professional	3.2 (1.5)	
Main diagnosis		
Schizophrenia and other psychotic disorders	2.3 (1.8)	0.784 ^b^
Bipolar disorder	2.3 (1.8)	
Depressive disorders	1.8 (1.6)	
Personality disorder	2 (1.4)	
Obsessive-compulsive disorder	1.3 (1.5)	

F&V: fruit and vegetable; VT: Vocational Training; ADL: Activities of Daily Living; ^a^: Student’s t test; ^b^: Anova * *p* < 0.05.

**Table A2:** Pearson’s correlation between sociodemographic and clinical variables and F&V intake.

	F&V	Age	Weight	BMI	FAST	SCIP-S	Subjective Well-Being
	Servings						
Age (years)							
*r*	0.185	1	−0.107	0.079	0.028	0.436	0.036
*p*-value	0.115		0.369	0.509	0.812	<0.001 ***	0.765
Weight (kg)							
*r*	−0.158	−0.107	1	0.852	0.071	−0.002	0.159
*p*-value	0.184	0.369		<0.001 ***	0.554	0.99	0.192
BMI (Kg/m^2^)							
*r*	−0.098	0.079	0.852	1	0.087	0.083	0.194
*p*-value	0.411	0.509	<0.001 ***		0.469	0.508	0.111
FAST							
*r*	0.067	0.028	0.071	0.087	1	−0.121	0.087
*p*-value	0.571	0.812	0.554	0.469		0.329	0.468
SCIP-S							
*r*	0.114	0.436	−0.002	0.083	−0.121	1	−0.220
*p*-value	0.243	<0.001 ***	0.99	0.508	0.329		0.074
Subjective							
well-being							1
*r*	−0.035	0.036	0.159	0.194	0.087	−0.220	
*p*-value	0.77	0.765	0.192	0.111	0.468	0.074	

F&V, fruit and vegetable; BMI, body mass index; FAST, Functioning Assessment Short Test; SCIP-S, Screen for Cognitive Impairment in Psychiatry; *** *p* < 0.001.
